# The Use of a Scoring System to Guide Thromboprophylaxis in a High-Risk Pregnant Population

**DOI:** 10.1155/2011/652796

**Published:** 2011-09-08

**Authors:** D. Schoenbeck, A. Nicolle, K. Newbegin, J. Hanley, A. D. Loughney

**Affiliations:** ^1^Department of Women's Services and Royal Victoria Infirmary, Newcastle Hospitals NHS Foundation Trust, Newcastle upon Tyne NE1 4LP, UK; ^2^Department of Haematology and Royal Victoria Infirmary, Newcastle Hospitals NHS Foundation Trust, Newcastle upon Tyne NE1 4LP, UK

## Abstract

Guidelines for thromboprophylaxis in pregnancy are usually based upon clinical observations and expert opinion. For optimal impact, their use must be attended by consistency in the advice given to women. In this observational study, we evaluated the performance of a scoring system, used as a guide for clinicians administering dalteparin to pregnant women at increased risk of venous thromboembolism. The work included 47 women treated with dalteparin prior to adoption of the scoring system and 58 women treated with dalteparin after its adoption. The indication for thromboprophylaxis was recorded in each case together with details of the regimen employed, obstetric, and haematological outcomes. The main outcome measure was to determine whether consistency improved after adoption of the scoring system. We also recorded the occurrence of any new venous thromboembolism, haemorrhage, the use of regional anaesthesia during labour, evidence of allergy, and thrombocytopenia. We found that use of the scoring system improved the consistency of advice and increased the mean duration of thromboprophylaxis. None of the subjects suffered venous thromboembolism after assessment using the scoring system. There was no increase in obstetric or anaesthetic morbidity when dalteparin was given antenatally period and no evidence of heparin-induced thrombocytopenia.

## 1. Introduction

Venous thromboembolism (VTE) has been the commonest noniatrogenic, direct cause of maternal death in England and Wales for many years, reflecting a longstanding UK wide pattern of disease [1, 2]. This is largely attributable to pregnancy-related changes in clotting factors that lead to a state of physiological hypercoagulability [3, 4]. A number of additional factors that increase the risk of a woman developing VTE in pregnancy have been identified however, including a personal or family history of VTE, increased maternal age or body weight, and the presence of a known heritable thrombophilia [5–8]. 

Although an established VTE in pregnancy may be treated successfully with therapeutic doses of heparin, prevention is preferable to cure because of the high mortality and long-term morbidity associated with established disease [9, 10]. Low-molecular-weight heparins (LMWHs) are effective thromboprophylactic agents, do not cross the placenta, and do not appear in breast milk [11–14]. They can be used safely in pregnancy and in breastfeeding with no adverse fetal or neonatal effects. Furthermore maternal allergy, osteoporosis, and thrombocytopenia are rare even after prolonged use [15–17], while epidural and spinal anaesthesia are also thought to be safe for labour and delivery if at least 12 hours have elapsed since the last administration time of the drug [18]. Because of their efficacy and good safety profile, the use of low molecular weight heparins for thromboprophylaxis in pregnancy has increased greatly in recent years. 

A measured approach to the use of LMWH is nevertheless essential because although side effects are rare, their increased use in pregnancy is likely to be associated with an increase in the absolute numbers of adverse events encountered. In addition, self-administration of parenteral agents seems onerous to some women and in labour, regional anaesthesia may occasionally be withheld or delayed because LMWH has been administered in the preceding 12 hours. Bearing these issues in mind, we constructed a novel Thromboprophylaxis Scoring System in order to identify women at high risk of VTE in pregnancy, to guide the timing of onset and duration of therapy, and thereby to promote consistency in clinical practice. In the present study we compare the management of women with a perceived high risk of VTE before and after introduction of the scoring system, while documenting pregnancy outcomes in the two groups using a number of obstetric and haematological indices. We also comment upon the content of the scoring system in relation to recommendations subsequently published by the Scottish Intercollegiate Guidelines Network (SIGN, 2002) and by the Royal College of Obstetricians and Gynaecologists (RCOG, 2009) [19, 20].

## 2. Patients and Methods

### 2.1. Construction of the Scoring System

Thromboprophylaxis with the LMWH dalteparin was introduced into clinical practice in the Royal Victoria Infirmary, Newcastle upon Tyne, in 1996. Between January 1997 and April 2001, women perceived by the GP, Community Midwife or Obstetrician to be at high risk of VTE in pregnancy were referred to an Obstetric Medicine Clinic for specialist advice. Here, a full medical history was taken and a thrombophilia screen was performed if a personal or family history of VTE was elicited. The need for treatment with dalteparin, the gestation for initiation of the drug, and the total duration of prophylaxis was then gauged by a consultant physician and a consultant obstetrician on an individualised basis. No specific local guidelines were in place to promote referral to the clinic or to guide timing and duration of therapy, but an established principle was that when prophylaxis was being offered on the grounds of a VTE in a previous pregnancy, treatment should commence four weeks prior to the gestation of that event. 

In April 2001, a multidisciplinary group of physicians, haematologists, and obstetricians was convened to provide local guidance for thromboprophylaxis in pregnancy. This group came to a consensus opinion about the relative importance of individual risk factors for the development of VTE in pregnancy by drawing upon available evidence and personal experience. In particular, reference was made to articles published in the British Journal of Obstetrics and Gynaecology in 1999 and the British Journal of Haematology in 2001 [21, 22], the latter having been endorsed by the British Society for Haematology. Summaries of these papers are presented as Tables 1 and 2.

On these bases, a Thromboprophylaxis Scoring System was compiled in which individual risk factors were allocated a weighted score (Table 3). It was agreed that the total score allocated to each woman referred for review at the Obstetric Medicine Clinic thereafter would be used to determine the need for prophylaxis and the gestation at which prophylaxis should be commenced. After treatment was started, the aim was to continue until 6 weeks postpartum in all cases. In this way, it was hoped that a uniform high standard of evidence-based care could be provided to all women referred to the clinic. The risk factors identified on the scoring system were also promoted as a guide for referral to the clinic after being made available to GPs, community midwives, and other consultant obstetricians in the hospital.

### 2.2. Database Construction

A database was constructed containing the details of all women referred for specialist advice to the Obstetric Medicine Clinic from January 1997, who were deemed to be at an increased risk of VTE and who were therefore given dalteparin. Two groups of women were entered onto this database, the first group consisting of women treated with dalteparin prior to the introduction of the scoring system (1997–2001) and the second group consisting of women prescribed dalteparin after its introduction (2001–2003). The information recorded on the database included maternal age and weight at booking. Previous personal obstetric, medical, and surgical histories were also noted together with the use of other drugs, known allergies, and the presence of any relevant family history of VTE. The stated indication for thromboprophylaxis was then recorded, together with the gestation at which dalteparin was commenced, the dose administered, and the duration of therapy. Side effects of therapy were also documented, including evidence of local or systemic allergic reactions. Platelet counts were determined 7 days after therapy commenced then monthly thereafter. Each patient's lowest count was recorded on the database regardless of gestation. In addition, the results of thrombophilia screening performed before, during or after pregnancy, were recorded. Finally, obstetric notes were examined and a record was made of each woman's antenatal, intrapartum, and postnatal progress, including details of antepartum haemorrhage, gestation, and mode of delivery, estimated blood loss at delivery, blood transfusion, and the use of regional analgesia in labour or at caesarean section.

### 2.3. Exclusions

The Thromboprophylaxis Scoring System was constructed to assist in the care of women identified antenatally to be at an increased risk of a pregnancy-related VTE. In the period of study, low-molecular-weight heparin was also prescribed for women having an elective or emergency caesarean section but who had no additional risk factors for VTE, for a period of 1 to 5 days, with an increase in the duration of prophylaxis being advocated in the more recent years. In addition, women with a BMI over 35 kg/m^2^ and woman admitted to the antenatal and postnatal wards for in-patient care are also now risk assessed for VTE but this was not standard practice during the study period. Data from these groups of patients have not been included in this paper.

### 2.4. Statistical Analyses

Student's *t*-test was used to compare the mean values of continuous variables in the two groups while variance in the time of commencement of therapy was examined with reference to tables of *F*. For categorical data, 2 × 2 and 2 × *k* contingency tables were constructed then *χ* ^2^ analyses was performed.

## 3. Results

In the 52 months prior to introduction of the scoring system, 47 women commenced dalteparin therapy for thromboprophylaxis after referral to the Obstetric Medicine Clinic. Over this time course, 18 542 women gave birth in the unit. In the 32 months after its introduction, a further 58 women were treated with dalteparin after referral to the clinic, over which time 13 975 women gave birth in the unit. Over the whole study period, 91 women received both antenatal and postnatal thromboprophylaxis while the remaining 14 received postnatal prophylaxis alone. The proportion of women attending the hospital who were given thromboprophylaxis therefore increased after introduction of the scoring system (*P* < 0.001). The mean age [SD] of women given thromboprophylaxis before introduction of the scoring system was 28.9 [5.6] years compared to 29.9 [5.5] years after its introduction, representing no change (*P* > 0.05). The mean booking weight [SD] was 74.3 [17.0]  kg before the scoring system was introduced compared to 78.8 [16.3]  kg after, again representing no change (*P* > 0.05).

### 3.1. Previous History of Venous Thrombosis While Taking the Combined Oral Contraceptive Pill

Prior to introduction of the scoring system, 14 women were identified who had developed a VTE while taking the combined oral contraceptive pill. These included 4 women with a below knee thrombosis, 9 with a thrombosis at any venous site above the knee, and 1 woman with a pulmonary embolus. Only 2 of the group had a family history of VTE. Dalteparin prophylaxis was commenced at a gestation varying between 18 weeks gestation and full term, then continued until 4 to 6 weeks postpartum. After introduction of the scoring system, a further 19 women were identified with a history of pill-related VTE, including 7 with a below knee thrombosis, 10 with thrombosis above the knee, and 2 with a pulmonary embolus. Three had a family history of VTE. Thromboprophylaxis was commenced between 11 and 30 weeks gestation and continued until 6 weeks postpartum in each case.

The gestation at commencement of therapy was compared between the two groups in order to determine whether the consistency of advice changed after introduction of the scoring system. Women were only included in this analysis if they had suffered an above knee thrombosis or a pulmonary embolus while taking the pill, but had no other identified risk factors for VTE. This was to ensure like-for-like comparison between the two groups. In these circumstances, before introduction of the scoring system, thromboprophylaxis commenced between 18 and 40 weeks gestation with a mean starting point [SD] of 31.1 [7.7] weeks of gestation (*n* = 9). After the scoring system was introduced, therapy was commenced between 27 and 29 weeks of gestation, with a mean [SD] of 28.8 [0.47] weeks (*n* = 10). Although the mean gestation at which prophylaxis with dalteparin was commenced was unchanged by introduction of the scoring system (*P* > 0.05), variance in the timing of onset of therapy was significantly reduced (*P* < 0.001).

### 3.2. Previous History of Venous Thrombosis in Pregnancy

Between January 1997 and April 2001, 22 women were identified who gave a history of venous thrombosis in a previous pregnancy, including 3 whose presentation had been with thrombosis below the knee, 12 with thrombosis above the knee, and a further 7 presenting with pulmonary embolus. All of these events took place in the second or third trimester of pregnancy. In each case, prophylactic dalteparin was prescribed, commencing between 1 and 9 weeks prior to the gestation at which the previous thrombosis had been identified. Between May 2001 and December 2003, a further 23 women were identified who gave a history of venous thrombosis in a previous pregnancy, including 3 women with a history of below knee thrombosis, 15 with a previous above knee thrombosis, and 5 women with a previous pregnancy related pulmonary embolus. Nineteen of these events took place in the second and third trimesters and four took place in the puerperium. In each case, a prophylactic dose of dalteparin was given, commencing between 4 and 22 weeks prior to the gestation of occurrence of the previous VTE, but not later than 30 weeks gestation in any single case.

In general, earlier anticoagulation was advised after introduction of the scoring system (26.7 [6.4] weeks *versus* 20.7 [7.7] weeks, *P* < 0.01). Variance analysis could not be employed to examine the consistency of advice given in this category due to the clinical heterogeneity encountered. Only 12/22 women commenced dalteparin at least 4 weeks prior to the gestation of the previous VTE before introduction of the scoring system however, compared to 23/23 after its introduction. This represents a significant change in practice (*P* < 0.001), suggesting greater consistency in clinical decision making.

### 3.3. Previous History of Unprovoked and Provoked Venous Thrombosis Outside of Pregnancy

Before introduction of the scoring system, 6 women were identified who had previously suffered a nonobstetric, unprecipitated VTE, including 2 women with a below knee VTE, and 4 with a pulmonary embolus. Two of the women who had previously had a pulmonary embolus also had a family history of VTE. Dalteparin was commenced between 18 and 38 weeks gestation then continued uniformly until 6 weeks postpartum. After introduction of the scoring system, another 7 women were identified with a previous unprovoked VTE including 1 with a below knee thrombosis, 2 with an above knee thrombosis, and 4 with a pulmonary embolus. Two of these had a family history of VTE. The timing of commencement of thromboprophylaxis varied between 14 and 28 weeks gestation after introduction of the scoring system and continued until 6 weeks postpartum in each case. 

To examine consistency of advice, the timing of onset of therapy was analysed in 4 women treated before introduction of the scoring system and 5 women treated after its introduction, none of whom had additional risk factors for VTE. The gestation [SD] at onset of treatment was 28.0 [9.3] weeks *versus* 28.0 [0.71] weeks, respectively, demonstrating greater consistency after introduction of the scoring system (*P* < 0.001). 

Over the whole period of study, 5 women received a six-week course of postpartum thromboprophylaxis on the basis of a previous provoked VTE. These included three women with a history of lower limb thrombosis following fracture of the femur and two women with a history of lower limb thrombosis following pelvic fracture. Although the numbers treated in this group were too small to allow meaningful statistical analysis, no clear change in clinical practice was noted after introduction of the scoring system*.*

### 3.4. Other Indications for Thromboprophylaxis

Three women received dalteparin on the basis of a history of VTE in at least two first- or second-degree relatives before introduction of the scoring system. Each had additional risk factors; two were heterozygous for Factor V Leiden, while the third recorded a booking weight of 124 kg and was 36 years of age. In each case, prophylaxis was commenced between 32 and 40 weeks of gestation and was continued for 6 weeks into the puerperium. 

After introduction of the scoring system, 6 women with a strong family history of VTE received thromboprophylaxis. Three were heterozygous for Factor V Leiden, one had protein S deficiency, one had a booking weight of 135 kg and the final case was of a woman who was 41 years of age and had a booking weight of 102 kg. 

In general, although the groups were heterogeneous in nature, the mean gestation [SD] at commencement of therapy was unchanged after introduction of the scoring system (37.3 [4.6] weeks versus 37.0 [6.0] weeks, *P* > 0.05). All women in this category received prophylactic dalteparin from delivery until 6 weeks postpartum.

### 3.5. Retrospective Application of the Thromboprophylaxis Scoring System

For each of the 47 women given thromboprophylaxis before introduction of the scoring system, a score was calculated retrospectively by relating each individual's risks to the scoring system table. The difference between actual timing of onset of therapy and the timing that would have been suggested with reference to the scoring system was then calculated. Earlier onset of therapy would have been advised in 30 cases, ranging from 1 to 28 weeks of additional heparinisation. Later onset of therapy would have been advised in 10 cases, ranging from 2 to 16 weeks less heparinisation. An identical timing of onset of therapy would have been advised in the remaining 7 cases. In general, the mean onset of timing of therapy would have been reduced from 29.7 [7.4] weeks to 24.9 [8.5] weeks, representing a significantly earlier onset of therapy (*P* < 0.01).

### 3.6. Occurrence of New Thromboses

None of the women in the present study suffered a VTE in pregnancy or the puerperium after assessment in the Obstetric Medicine Clinic, before or after introduction of the scoring system.

### 3.7. Analgesia

Of the 91 women given antenatal thromboprophylaxis, 85 laboured before delivery and 39 of these had an epidural sited for analgesia. Of the 14 women receiving exclusively postpartum thromboprophylaxis, 12 laboured before delivery and of these, 6 received an epidural in labour. In each case, at least twelve hours had elapsed since administration of dalteparin and no neurological or haemorrhagic complications were encountered. The use of antenatal thromboprophylaxis therefore did not mitigate against the use of epidural for analgesia in labour (*P* > 0.05).

### 3.8. Mode of Delivery

The mode of delivery for women in the study is shown in Table 4. Women who received antenatal thromboprophylaxis were at neither an increased nor a decreased risk of instrumental vaginal or caesarean delivery compared to women who received postpartum thromboprophylaxis alone (*P* > 0.05).

### 3.9. Obstetric Haemorrhage

Between 1997 and 2003, 4/91 women who received antenatal prophylaxis with dalteparin were admitted to hospital because of antepartum haemorrhage. Three women suffered unexplained spotting of blood from the vagina in the third trimester that settled spontaneously, while one woman experienced a major placental abruption requiring emergency caesarean section at full term. Of the 14 women given exclusively postpartum prophylaxis, only one experienced a significant antepartum haemorrhage. In this case, the patient developed severe pre-eclampsia then suffered a placental abruption at 30 weeks of gestation, requiring delivery by caesarean section. Dalteparin therapy was given for 6 weeks after delivery as previously planned. The use of dalteparin before delivery was not associated with a change in the rate of antepartum haemorrhage (*P* > 0.05).

The use of antenatal dalteparin did not alter the mean estimated blood loss at spontaneous vaginal, instrumental, or caesarean delivery (Table 4). One woman receiving exclusively postnatal thromboprophylaxis required blood transfusion after giving birth by caesarean section while five women who had been given antenatal dalteparin were transfused. These included four who underwent emergency caesarean section for obstetric indications and one woman with a prolonged second stage of labour who was delivered by forceps. Despite these observations, the use of blood transfusion for women given antenatal thromboprophylaxis was not increased to a statistically significant extent (Fisher's Exact Test, *P* = 0.89).

### 3.10. Allergy

Throughout the period of study dalteparin was well tolerated although three women developed itch and a localised macular rash shortly after commencing therapy. In each case, dalteparin was replaced by tinzaparin, resulting in a rapid and complete resolution of symptoms. No other allergic reactions were noted.

### 3.11. Thrombocytopenia

Fourteen women receiving antenatal dalteparin recorded a platelet count below 150 × 10^9^/L during the study period but only three recorded a count below 100 × 10^9^/L, with minimum values of 98, 88, and 72 × 10^9^/L, respectively. In each of these cases there was clear evidence of pre-eclampsia. The fall in platelet count led to a temporary discontinuation of therapy but dalteparin was recommenced postpartum and thereafter, platelet counts recovered despite ongoing use of the drug.

### 3.12. Thrombophilia Screening

The thrombophilia screen included functional chromogenic assays for antithrombin, Protein C and Protein S. Activated Protein C resistance was assessed with molecular confirmation of Factor V Leiden when indicated. Prothrombin gene G20210A identification was also undertaken together with lupus anticoagulant and anticardiolipin antibody status definition. Screening was carried out either before or during pregnancy, before dalteparin was commenced. Low Protein C or Protein S levels identified during pregnancy were confirmed by repeat assay several weeks after stopping treatment. An array of heritable thrombophilias was identified (Table 5). 

Prior to introduction of the scoring system, 39 of the 47 women with a personal or family history of VTE were screened for heritable thrombophilias, compared to all 58 women given dalteparin after its introduction. This represents a significant increase in the rate of screening (*P* < 0.01).

## 4. Discussion

The authors of a 2002 Cochrane Review entitled Prophylaxis for Venous Thromboembolic Disease in Pregnancy and the Early Postnatal Period stated that there was insufficient evidence available upon which to base firm recommendations for thromboprophylaxis in pregnancy [23]. Robust research was called for to inform clinical practice, mirroring the position taken a number of years earlier by similar authors [24]. With evidence of significant morbidity and mortality arising from VTE in pregnancy however, including case reports amassed through over 50 years of Confidential Enquiries reports, SIGN issued firm guidance for thromboprophylaxis in pregnancy in 2002 [19], necessarily basing their recommendations largely upon expert opinion. Here, it was suggested that women with unprovoked, pill-related or pregnancy-related previous VTE should be offered prophylaxis with a low-molecular-weight heparin as early in pregnancy as possible and that therapy should continue into the puerperium. This move towards early anticoagulation was endorsed by the RCOG in their 2004 Green-top Guideline document, “Thromboprophylaxis During Pregnancy, Labour and After Vaginal Delivery” [25], now superseded by the 2009 Green-top Guideline “Reducing the risk of thrombosis and embolism during pregnancy and the puerperium” [20]. Audit data collected through the Confidential Enquiries system in coming years will provide some measure of the success or failure of these guidelines, particularly in the face of current trends towards an increase in maternal age, maternal obesity, and operative delivery currently being experienced in the UK. 

The SIGN and RCOG Guidelines make a limited attempt to assess an individual's risk for VTE and to tailor the therapeutic regimen offered accordingly. This simplified approach is justified if (1) the risk of a VTE arising at any point in time in a high-risk population remains similar throughout each trimester of pregnancy, (2) prophylaxis with low-molecular-weight heparin is effective in preventing VTE throughout pregnancy, (3) potential adverse events associated with the prolonged use of prophylactic low molecular weight heparin are outweighed by the benefits of therapy, and (4) the simplicity of advice offered lends itself to consistency in clinical practice. In most respects, these criteria have already been met. For example, although some reports suggest that the absolute risk of new or recurrent VTE during pregnancy is low [26, 27], one large cohort study bears out the principle that the risk of recurrent VTE is nevertheless increased throughout pregnancy when thromboprophylaxis is withheld, with a particularly high-risk manifest postpartum [28]. Furthermore, there have been several case series reported in which the prolonged use of low-molecular-weight heparin in pregnancy appeared to be effective in preventing thrombosis while causing few clinically significant side effects. It remains to be shown however that use of the SIGN or RCOG Guidelines promotes consistency in clinical practice. This is an important goal if their full potential benefit is to be realised. 

Introduction of the Thromboprophylaxis Scoring System presented in this paper brought about a marked improvement in the consistency of the clinical management of women at increased risk of VTE in pregnancy. A theoretical advantage of the scoring system over the SIGN and RCOG Guidelines is that it has achieved this consistency while retaining a highly individualised assessment of a woman's VTE risk in the face of multiple risk factors for thromboembolism. Whether or not this delivers a clinical advantage remains unproven. To this end, the scoring system could now be used in the context of a randomised controlled trial in the manner recommended in the Cochrane Review of 2004 [24]. Alternatively, if the pragmatic opinion of authors of the SIGN and RCOG documents is accepted together with the evidence that the Thromboprophylaxis Scoring System improves consistency of approach, the recommendations held within the system might now be altered to comply with the recommendations of these guidelines. Specifically, a score of 2.0 could trigger prophylaxis with a standard, once daily dose of LMWH throughout pregnancy while a score of 3.0 or more could for example trigger weight adjusted prophylaxis with concomitant anti-Xa monitoring. 

In summary, use of the Thromboprophylaxis Scoring System presented in this paper improved consistency of approach when advice was being given to women with a high risk of VTE in pregnancy. Although specific elements of the advice given would require modification to comply with current SIGN and RCOG Guidelines, the scoring system remains a useful tool in clinical practice and could be employed in further large-scale research work examining the efficacy of different thromboprophylaxis regimens in high-risk pregnancies.

## Figures and Tables

**Table 1:** Anticoagulation recommendations published in the BJOG, August 1999 [21].

*Very high risk*		
Previous VTE while taking anticoagulants	Thromboprophylaxis with dose adjusted for anti-Xa activity throughout pregnancy and for 12 weeks postpartum.	
VTE in the current pregnancy		
Antithrombin deficiency		

*High risk*		
Previous VTE while not on anticoagulants	Fixed dose of LMWH from 24 weeks, earlier if additional risk factors for VTE. With previous pregnancy associated VTE, start 4–6 weeks ahead of gestation of the previous event and continue for 12 weeks postpartum.	
Protein C deficiency and FMH		
Homozygous Factor V Leiden		
Homozygous prothrombin gene mutation		
Combined thrombophilias		

*Moderate risk*		
Heterozygous factor V Leiden	Postpartum LMWH prophylaxis for 6 weeks.	
Heterozygous prothrombin gene mutation		
Heterozygous protein S deficiency		
FMH alone		

*Relatively low risk*		
Heterozygous Factor V Leiden with no FMH	Monitor for additional risks.	
Heterozygous PT gene mutation with no FMH		

VTE: venous thromboembolism, LMWH: low molecular weight heparin, FMH: family history of VTE.

**Table 2:** Anticoagulation recommendations published in the British Journal of Haematology, January 2001 [22].

*High risk*		
On long-term anticoagulants	Pregnancy-long thromboprophylaxis. Consider using 75 anti Xa units/kg 12 hourly. Continue treatment for 6 weeks postpartum.	
Type 1 antithrombin deficiency		
Type 2 reactive site antithrombin defect		

*Moderate risk*		
Previous precipitated VTE plus thrombophilic defect	Consider antenatal thromboprophylaxis with 4000 to 5000 anti-Xa units of LMWH once daily, starting in either the first or second trimester and continuing for 6 weeks postpartum.	
Previous unprecipitated VTE		
Homozygous factor V Leiden plus FMH		
Homozygous prothrombin gene mutation plus FMH		
Heterozygous protein C deficiency plus FMH		
Combination of thrombophilias		

*Slightly increased risk*		
Heterozygous protein S plus FMH	Consider thromboprophylaxis after delivery for 6 weeks, particularly if other risk factors are present such as increased maternal age.	
Heterozygous factor V Leiden plus FMH		
Heterozygous prothrombin gene mutation plus FMH		
Previous precipitated VTE, no thrombophilia		

VTE = venous thromboembolism, LMWH = low molecular weight heparin, FMH = family medical history of VTE.

**Table 3:** The Thromboprophylaxis Scoring System.

Risk factor	Score
Age >35 years	0.5
Weight >120 kg	0.5
VTE in two or more first or second degree relatives	0.5
Previous nonobstetric provoked VTE	1.0
Previous nonobstetric unprovoked VTE	2.0
Previous VTE on the combined oral contraceptive pill	2.0
Previous obstetric VTE*	2.0
Antithrombin deficiency (use anticoagulant dose adjusted for weight)	3.0
Protein C deficiency**	1.5
Protein S deficiency**	1.0
Factor V Leiden**	1.0
Prothrombin gene mutation (G20210A)**	1.0
Antiphospholipid antibodies***	1.0

Total Score	

Total score/Thromboprophylaxis: Under 1.0: conservative management, lifestyle advice; 1.0–1.5: from delivery until 6 weeks postpartum; 2.0–2.5: from 28 weeks until 6 weeks postpartum; 3.0 or more: from diagnosis of pregnancy until 6 weeks postpartum; *begin therapy at least 4 weeks before the gestation of the previous VTE; **pregnancy long therapy for homozygous conditions; ***individualised care for recurrent pregnancy loss.

VTE: venous thromboembolism. 'Precipitated' refers to VTE following an identifiable cause such as trauma, surgery, immobilisation, air travel or malignant disease.

**Table 4:** Mode of delivery and estimated blood loss.

	SVD	IVD	CS
Antenatal and postnatal dalteparin			
EBL [SD] mL	243 [102]	423 [186]	597 [292]
*n*	57	19	15

Postnatal dalteparin alone			
EBL [SD] mL	290 [263]	365 [42]	355 [132]
*n*	5	5	4

*P*	0.20	0.25	0.22

EBL: estimated blood loss; SVD: spontaneous vaginal delivery; IVD: instrumental vaginal delivery, incorporating all ventouse and forceps births; CS: caesarean sections, incorporating elective and emergency procedures; *n*: number of women in each group.

**Table 5:** Results of thrombophilia screening in women receiving dalteparin during or after pregnancy.

Indication for prophylaxis	APA	AT	PC	PS	FVL	PT	unscreened
previous VTE on COCP	0	0	0	1	6	1	3
previous VTE in pregnancy	3	0	1	1	3	3	0
previous unprovoked VTE	0	0	0	0	0	1	0
previous provoked VTE	0	0	0	0	0	0	0
other indications	0	0	0	1	5	0	0

VTE: venous thromboemboloism; COCP: combined oral contraceptive pill; APA: antiphospholipid antibodies; AT: antithrombin deficiency (untyped); PC: Protein C deficiency; PS: Protein S deficiency; FVL: heterozygous Factor V Leiden; PT: heterozygous prothrombin gene G20210A mutation. All patients were screened after introduction of the scoring system.

## References

[1] Walker AL, Wrigley AJ, Organe GS, Chamberlain RN, Martin WJ (1960). Pulmonary embolism. *Reports on Public Health and Medical Subjects Number 103. Report on Confidential Enquiries into Maternal Deaths in England and Wales 1955–1957*.

[2] Drife J, Lewis G (2007). Thrombosis and thromboembolism. *Saving Mothers’ Lives 2003–2005. Confidential Enquiry into Maternal and Child Health*.

[3] Clark P, Brennand J, Conkie JA, McCall F, Greer IA, Walker ID (1998). Activated protein C sensitivity, protein C, protein S, coagulation in normal pregnancy. *Thrombosis and Haemostasis*.

[4] Kjellberg U, Andersson NE, Rosen S, Tengborn L, Hellgren M (1999). APC resistance and other haemostatic variables during pregnancy and the puerperium. *Thrombosis and Haemostasis*.

[5] McColl MD, Ramsay JE, Tait RC (1997). Risk factors for pregnancy associated venous thromboembolism. *Thrombosis and Haemostasis*.

[6] Rosendaal FR (1999). Venous thrombosis: a multicausal disease. *The Lancet*.

[7] Weiss N, Bernstein PS (2000). Risk factor scoring for predicting venous thromboembolism in obstetric patients. *The American Journal of Obstetrics & Gynecology*.

[8] McColl MD, Walker ID, Greer IA (1999). The role of inherited thrombophilia in venous thromboembolism associated with pregnancy. *The British Journal of Obstetrics and Gynaecology*.

[9] Lindhagen A, Bergqvist A, Bergqvist D, Hallbook T (1986). Late venous function in the leg after deep venous thrombosis occurring in relation to pregnancy. *The British Journal of Obstetrics and Gynaecology*.

[10] Bergqvist A, Bergqvist D, Lindhagen A, Matzsch T (1990). Late symptoms after pregnancy related deep venous thrombosis. *The British Journal of Obstetrics and Gynaecology*.

[11] Nelson-Piercy C, Letsky EA, de Swiet M (1996). Low molecular weight heparin for obstetric thromboprophylaxis: experience of 69 pregnancies in 61 high risk women. *The American Journal of Obstetrics & Gynecology*.

[12] Ellison J, Walker ID, Greer IA (2000). Antenatal use of enoxaparin for prevention and treatment of thromboembolism in pregnancy. *The British Journal of Obstetrics and Gynaecology*.

[13] Matzsch T, Bergqvist D, Bergqvist A (1991). No transplacental passage of standard heparin or an enzymatically depolymerised low molecular weight heparin. *Blood Coagulation &amp; Fibrinolysis*.

[14] Richter C, Sitzmann J, Lang P, Weitzel H, Huch A, Huch R (2001). Excretion of low molecular weight heparin in human milk. *The British Journal of Clinical Pharmacology*.

[15] Harenberg J, Hoffmann U, Huhle G, Winkler M, Bayerl C (2001). Cutaneous reactions to anticoagulants recognition and management. *The American Journal of Clinical Dermatology*.

[16] Shefras J, Farquharson RG (1996). Bone density studies in pregnant women receiving heparin. *European Journal of Obstetrics Gynecology and Reproductive Biology*.

[17] Warkentin TE, Levine MN, Hirsh J (1995). Heparin induced thrombocytopoenia in patients treated with low molecular weight heparin or unfractionated heparin. *The New England Journal of Medicine*.

[18] Bergqvist D, Lindblad B, Matzsch T (1992). Low molecular weight heparin for thromboprophylaxis and epidural/spinal anaesthesia. Is there a risk?. *Acta Anaesthesiologica Scandinavica*.

[19] Scottish Intercollegiate Guidelines Network (1999). *Pregnancy and the Puerperium: Antithrombotic Therapy*.

[20] Royal College of Obstetricians and Gynaecologists (2009). *Reducing the Risk of Thrombosis and Embolism during Pregnancy and the Puerperium*.

[21] McColl MD, Walker ID, Greer IA (1999). The role of inherited thrombophilia in venous thromboembolism associated with pregnancy. *The British Journal of Obstetrics and Gynaecology*.

[22] Waker ID, Greaves M, Preston FE (2001). Guideline: investigation and management of heritable thrombophilia. *The British Journal of Haematology*.

[23] Gates S, Brocklehurst P, Davis LJ (2002). Prophylaxis for venous thromboembolic disease in pregnancy and the early postnatal period. *Cochrane Database of Systematic Reviews*.

[24] Brocklehurst P (1997). Future research needs for venous thrombo-embolic disease in obstetrics and gynaecology. *Bailliere’s Clinical Obstetrics and Gynaecology*.

[25] Royal College of Obstetricians and Gynaecologists (2004). *Thromboprophylaxis during Pregnancy, Labour and after Vaginal Delivery*.

[26] Brill-Edwards P, Ginsberg JS, Gent M (2000). Safety of withholding heparin in pregnant women with a history of venous thromboembolism. Recurrence of clot in this pregnancy study group. *The New England Journal of Medicine*.

[27] Heit JA, Kobbervig CE, James AH, Petterson TM, Bailey KR, Melton LJ (2005). Trends in the incidence of venous thromboembolism during pregnancy or postpartum: a 30-year population-based study. *Annals of Internal Medicine*.

[28] Pabinger I, Grafenhofer H, Kaider A (2005). Risk of pregnancy-associated recurrent venous thromboembolism in women with a history of venous thrombosis. *Journal of Thrombosis and Haemostasis*.

